# Does a modified STarT Back Tool predict outcome with a broader group of musculoskeletal patients than back pain? A secondary analysis of cohort data

**DOI:** 10.1136/bmjopen-2016-012445

**Published:** 2016-10-14

**Authors:** J C Hill, E K Afolabi, M Lewis, K M Dunn, E Roddy, D A van der Windt, N E Foster

**Affiliations:** Institute of Primary Care and Health Sciences, Keele University, Staffordshire, UK

**Keywords:** prognosis, prospective cohort, STarT Back Tool, Risk stratification

## Abstract

**Objectives:**

The STarT Back Tool has good predictive performance for non-specific low back pain in primary care. We therefore aimed to investigate whether a modified STarT Back Tool predicted outcome with a broader group of musculoskeletal patients, and assessed the consequences of using existing risk-group cut-points across different pain regions.

**Setting:**

Secondary analysis of prospective data from 2 cohorts: (1) outpatient musculoskeletal physiotherapy services (PhysioDirect trial n=1887) and (2) musculoskeletal primary–secondary care interface services (SAMBA study n=1082).

**Participants:**

Patients with back, neck, upper limb, lower limb or multisite pain with a completed modified STarT Back Tool (baseline) and 6-month physical health outcome (Short Form 36 (SF-36)).

**Outcomes:**

Area under the receiving operator curve (AUCs) tested discriminative abilities of the tool's baseline score for identifying poor 6-month outcome (SF-36 lower tertile Physical Component Score). Risk-group cut-points were tested using sensitivity and specificity for identifying poor outcome using (1) Youden's J statistic and (2) a clinically determined rule that specificity should not fall below 0.7 (false-positive rate <30%).

**Results:**

In PhysioDirect and SAMBA, poor 6-month physical health was 18.5% and 28.2%, respectively. Modified STarT Back Tool score AUCs for predicting outcome in back pain were 0.72 and 0.79, neck 0.82 and 0.88, upper limb 0.79 and 0.86, lower limb 0.77 and 0.83, and multisite pain 0.83 and 0.82 in PhysioDirect and SAMBA, respectively. Differences between pain region AUCs were non-significant. Optimal cut-points to discriminate low-risk and medium-risk/high-risk groups depended on pain region and clinical services.

**Conclusions:**

A modified STarT Back Tool similarly predicts 6-month physical health outcome across 5 musculoskeletal pain regions. However, the use of consistent risk-group cut-points was not possible and resulted in poor sensitivity (too many with long-term disability being missed) or specificity (too many with good outcome inaccurately classified as ‘at risk’) for some pain regions. The draft tool is now being refined and validated within a new programme of research for a broader musculoskeletal population.

**Trial registration number:**

ISRCTN55666618; Post results.

**Strengths and limitations of this study**

- First study to demonstrate that modified STarT Back Tool items are similarly predictive of 6-month physical health across different musculoskeletal pain regions.
- Within two large independent cohorts it was consistently shown that a modified STarT Back Tool similarly predicts 6-month physical health outcome in other musculoskeletal pain regions as well as low back pain.
- A limitation of the study was that the original STarT Back Tool was not included in these two data sets, so a direct comparison between the performance of the original and modified STarT Back Tool versions for patients with low back pain was not possible.

## Introduction

The Keele STarT Back Tool is designed to stratify patients with low back pain according to their risk of future physical disability, in order that prognostic subgroups can receive matched treatment.^1^ For example, individuals at a low risk of persistent disabling problems can be reassured and discouraged from receiving unnecessary treatments and investigations, while those at high risk can matched to treatment which combines physical and psychological approaches.^2–4^ A large randomised trial testing a risk stratification approach (use of the STarT Back Tool and matched treatments) for low back pain in comparison to best current care demonstrated superior clinical and cost outcomes.^5^ In addition, an implementation study testing risk stratification for patients with low back pain in routine general practice demonstrated significant improvements in physical function and time off work, sickness certification rates and reductions in healthcare costs compared to usual non-stratified care.^2^ Since low back pain accounts for only 17% of all UK primary care musculoskeletal consultations in general practice,^6^ if a similar screening tool could be used for patients with other common pain presentations, such as neck pain and knee pain, then there could be potential for stratified care to make a greater impact for patients and healthcare services.

A previous systematic review of 45 cohort studies^7^ reported that prognostic factors are often similar across different musculoskeletal presentations, with 11 factors predicting poor outcome at follow-up for at least two different musculoskeletal pain problems. Other studies have similarly shown that a generic set of baseline factors (pain intensity, episode duration, pain interference, depression and comorbid pain problems) predicts risk of a poor outcome across different pain regions, including back pain, headache, facial pain and knee pain, regardless of the specific location of pain or underlying pathology.^8–12^ These studies indicate that it might be possible to use the same prognostic factors as those included within the STarT Back Tool to discriminate risk status for a much larger group of musculoskeletal pain patients than those consulting with low back pain. The key benefit of using a single tool to stratify patients with a wide range of musculoskeletal conditions rather than multiple site-specific prognostic screening tools is its simplicity for use in busy clinical practice.

While the likely value and acceptability of extending risk stratification to patients with other common musculoskeletal pain is as yet unknown, evidence suggests that the majority of general practitioners (GPs) consider prognosis to be important in their clinical decision-making for musculoskeletal treatment.^13^ Despite the widespread support for prognostic information, the clinical reality is that predicting outcome in these patients is not always easy and patient's risk status is not typically included within medical records.^14^ GPs are not alone in wanting information about patients' likely prognosis over time, as >80% of musculoskeletal patients also want prognostic information from their GP, although less than a third actually receive this information.^14^ Existing musculoskeletal prognostic tools are available (eg, Linton and Hallden^15^ and Von Korff *et al*).^16^ ^17^ However, these prognostic tools were not designed or tested to support clinical decisions in primary care about matched treatments (stratified care); only the STarT Back Tool has been specifically developed and tested to guide patient treatment matching.

The aim of this study was therefore to investigate the performance of a modified STarT Back Tool for predicting future physical health outcome for a broader group of musculoskeletal pain patients. Specific objectives were to compare the predictive performance of a modified STarT Back Tool for patients with musculoskeletal pain in different body regions and assess the consequences (false-positive and false-negative rates) of using existing STarT Back Tool score cut-points for classifying patients as medium/high risk across different pain regions (neck, back, upper limb, lower limb and multisite pain).

## Methods

### Design

This study involved prespecified further analysis of existing data sets from two prospective cohorts of adults with musculoskeletal conditions consulting in two different services in the National Health Service, UK. Full ethical approval for both these studies was obtained and patients provided written informed consent prior to their research participation.

### Patient population

1. The PhysioDirect trial included 2249 adult musculoskeletal patients taking part in a randomised trial comparing a PhysioDirect service (telephone-based physiotherapy assessment and advice) with usual physiotherapy care.^18–20^ Primary outcome data (physical health measured using the SF-36v2 physical component score) at 6-month follow-up and baseline modified STarT Back Tool score were available for 1887 patients (84%) and were included in this analysis. The trial was conducted in four NHS community physiotherapy services in four different areas of England (Bristol, Somerset, Stoke-on-Trent and Cheshire). Adults (aged ≥18 years) who were referred by 94 GPs (covering a wide range of geographical areas and populations), or who referred themselves for physiotherapy for a musculoskeletal problem, were eligible for the trial. Patients completed postal questionnaires at baseline and 6 months after randomisation. Details about the PhysioDirect patient sample have been published.^18^ For the study reported here, we used patients from the control and intervention arms.
2. The SAMBA study was an observational cohort of adults attending an NHS musculoskeletal clinical assessment and treatment service at the primary–secondary care interface.^21^ ^22^ The study population included 2166 patients referred from primary care and subsequently triaged to musculoskeletal and back pain interface clinics in Stoke-on-Trent Primary Care Trust (PCT) over a 12-month period. Primary outcome data at 6-month follow-up (physical health measured using the SF-36v2 physical component score) and the modified STarT Back Tool score at baseline were available for 1082 patients (50%) who formed the study population for this evaluation. All adults (aged ≥18 years) capable of giving written informed consent were eligible to participate in the study. Patients completed study questionnaires before their first appointment during which consent was obtained and 6 month after that initial clinic appointment. Details of the SAMBA study sample have been published.^22^

### Modifying the STarT Back Tool

The original STarT Back Tool includes nine items of which five concern psychosocial factors (fear, catastrophising, anxiety, depression and bothersomeness). The PhysioDirect trial and SAMBA study included the STarT Back Tool's psychosocial items within their baseline questionnaires.^1^ These items were used without modification as they were developed from generic tools and are not specific to low back pain. However, the four further items of the original STarT Back Tool that capture three physical factors (referred pain from the back down the leg, comorbid pain in the neck and shoulder, and physical function with walking and dressing items) are specific to low back pain and therefore these items in their original form needed to be replaced by similar items that were applicable for all musculoskeletal patients. We therefore used proxy items for these outcome domains that were available in both data sets. The STarT Back Tool's two ‘function’ items (walking and dressing) were replaced by items from the generic EQ-5D^23^ (‘I have some problems in walking about’, Y/N and ‘I have some problems washing or dressing myself’, Y/N), and we used item 7 from the SF-12^24^ (‘How much bodily pain have you had?’ with positive responses defined as ‘extremely’ or ‘very severe’) instead of the original STarT Back Tool item for comorbid pain in the neck or shoulder. It was not possible to replace ‘referred pain from the back down the leg’ with an item that was suitable for all musculoskeletal pain and so this construct of the ‘spread of pain’ was omitted from the modified tool. To score the modified STarT Back Tool, responses from these eight items were summed (range 0–8) for all patients in both data sets. The original STarT Back Tool cut-off of 0–3 positive items was used to classify patients as at low risk and 4 or more as at medium or high risk. There were no reference standards for psychological distress in either the PhysioDirect or SAMBA data sets and so in this analysis we did not seek to examine the ability of the modified STarT Back Tool to identify a high-risk-only group. We believe that there is a strong clinical rationale for identifying musculoskeletal cases that are ‘at risk’ of a poor prognosis, which reflects the combined medium-risk and high-risk subgroup. In our previous IMPaCT Back study^2^ implementing risk stratification in general practice, the clinicians used a 6-item STarT Back Tool which only discriminated between low-risk and a combined medium-risk/high-risk group to decide which patients to refer or not to refer physiotherapy. In that study, the physiotherapists who received ‘at risk’ patients then used the full 9-item STarT Back Tool to discriminate the distressed patients who needed a psychologically informed physiotherapy treatment approach.

### Defining the body regions of pain

Participants were asked to indicate the primary site of their musculoskeletal pain for which they had sought treatment. From this information, patients were categorised as having one of the following regional pain problems: neck, back (thoracic or lumbar), upper limb, lower limb or multisite pain (pain in more than one region).

### Defining physical health outcome

The standardised summary score for the Physical Component Score (PCS) of the Short Form 36 (SF-36) Health Survey is population normalised (0 is worst physical health and 100 is best physical health) and was classified by tertiles (≤33, 34–66, >66) as has been used previously^25^ ^26^ with a 6-month poor outcome defined using the most severe tertile (≤33). Outcome was defined as poor physical health at 6-month follow-up using the SF-36 PCS because this was the most appropriate physical function outcome score available in both studies, and it has demonstrated good validity and responsiveness in this population.^27–29^

### Statistical analysis

All analyses were conducted separately for the two data sets and a descriptive comparison of the modified baseline STarT Back Tool scores (mean and SD) and proportion with poor 6-month physical health outcome (SF-36 PCS ≤33) calculated. Descriptive statistics using means and SDs were used to examine the modified STarT Back Tool score's distribution and investigate potential floor or ceiling effects (>10% of either lowest or maximum score).^30^

Predictive performance (discrimination) was assessed by calculating ROC curve AUCs for baseline modified STarT Back Tool total scores against 6-month poor physical health outcome (dichotomised as poor/good) for each of the five different bodily pain presentations and their equality compared using STATA's ‘roccomp’ command to establish whether AUC differences were statistically significant.

To examine whether the optimal subgroup cut-point on the modified STarT Back Tool total score to discriminate low from medium/high risk for poor 6-month physical health outcome was consistent across the five different pain regions and across the two data sets, we used two methods based on sensitivity and specificity of each potential cut-point. First, we used Youden's J Statistic which is calculated as sensitivity+specificity−1 for each potential cut-point and the optimal cut-point is the tool score with the highest value.^31^ ^32^ Second, we a priori agreed that specificity should not fall below 0.7, as lower values would mean potentially overtreating >30% of medium-risk/high-risk patients, which was considered an unacceptable level for an efficient matched treatment approach.

In this study, we were not able to identify optimal subgroup cut-points on the modified STarT Back Tool to distinguish between medium-risk and high-risk patients as there were no reference standards for psychological distress in the two available data sets. The original STarT Back Tool used these reference standards to identify distress ‘caseness’ at baseline, and identified the optimal cut-point to screen for these distressed ‘cases’ using a psychological subscale score. Without these reference standards for psychological distress, we were limited to determining optimal subgroup cut-points on the total scale score between low and medium/high risk alone.

## Results

### Distribution of the modified STarT Back Tool scores in both data sets

In the PhysioDirect trial sample (n=1887), the 8-item modified STarT Back Tool score at baseline was normally distributed with a mean (SD) of 3.35 (2.09); 8.4% had the lowest score (0) and 2.2% had the maximum score (8). The distribution of primary pain regions was reported by clinicians as: lower limb 31.1%, back 28.7%, upper limb 23.5%, neck 11.8% and multisite pain 4.8%. The 6-month SF-36 PCS mean (SD) was 43.7 (10.9) with 18.5% having a ‘poor outcome’ in their physical health at 6-month follow-up. The mean age was 48 years old and 60% were female.

In the SAMBA study sample (n=1082), the 8-item modified STarT Back Tool score at baseline was not normally distributed but had roughly equal numbers of all possible scores with a mean (SD) of 3.95 (2.65); 12.6% had the lowest score (0) and 10.9% had the maximum score (8). The distribution of primary pain sites was reported by patients as: lower limb 30.8%, back 26.7%, upper limb 23.8%, multisite pain 13.4% and neck 5.4%. The 6-month SF-36 PCS mean (SD) was 38.41 (12.76) with 28.2% having a ‘poor outcome’ in their physical health at 6-month follow-up. The mean age was 51 years old and 57% were female.

### Predictive performance of the modified STarT Back Tool score across pain regions in both data sets

**Figure 1:**
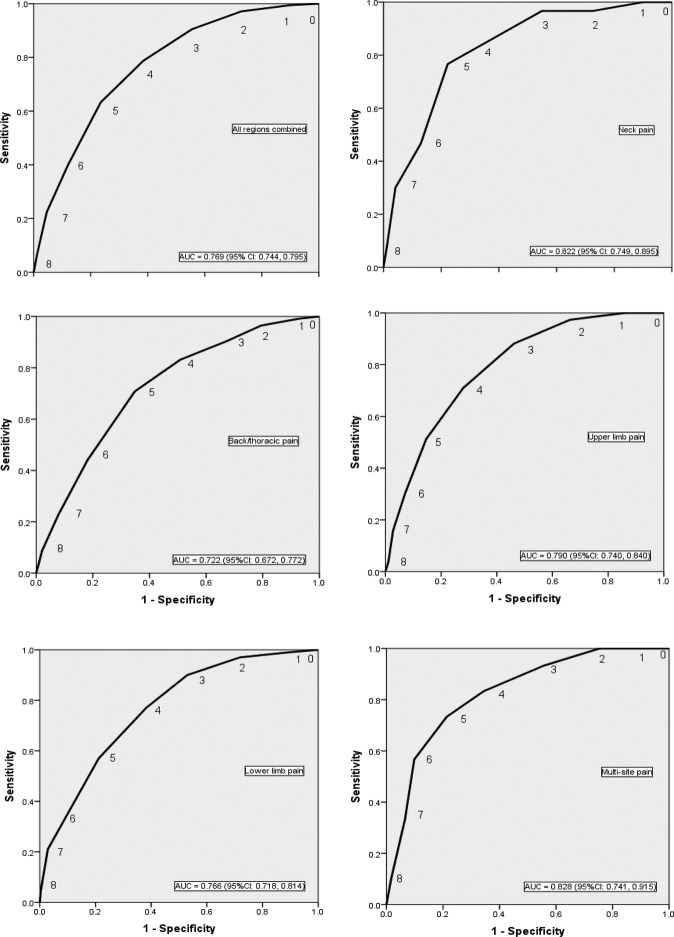
ROC curves for overall modified STarT Back tool scores against 6-month poor physical health outcome (SF-36 PCS ≤33) by different pain regions in the PhysioDirect data set. AUC, area under the receiving operator curve; ROC, receiver operating characteristic; SF-36, Short Form 36.

**Figure 2:**
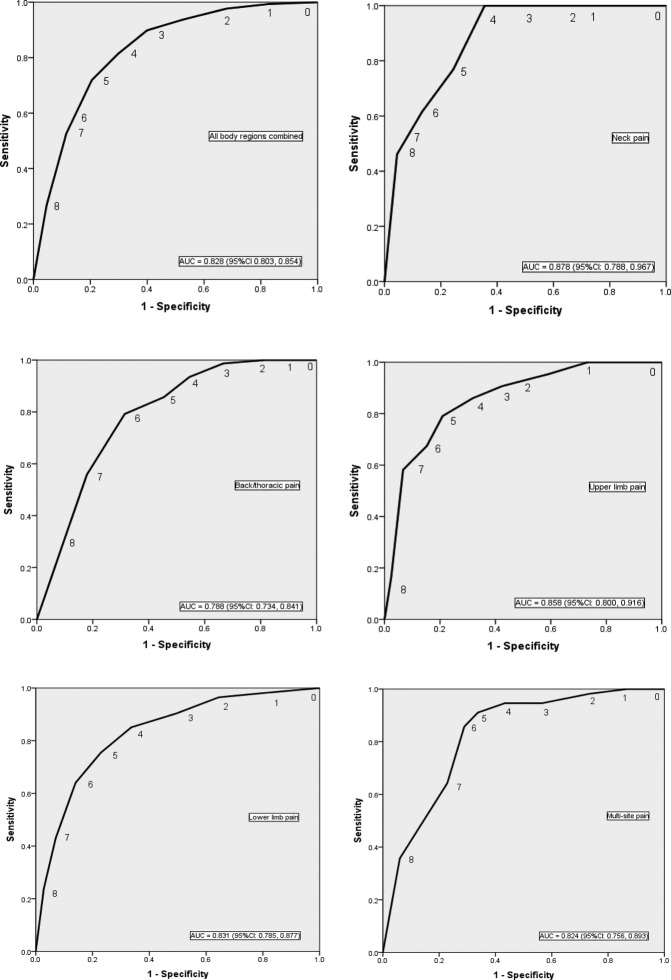
ROC curves for overall modified STarT Back tool scores against 6-month poor physical health outcome (SF-36 PCS ≤33) by different pain regions in the SAMBA data set. AUC, area under the receiving operator curve; ROC, receiver operating characteristic; SF-36, Short Form 36.

Predictive performance of the modified STarT Back Tool as determined by ROC curve AUCs ranged from 0.72 to 0.83 and was not found to be statistically different across different pain regions in the PhysioDirect trial (p=0.098) and SAMBA study (p=0.130) (presented in figures 1 and 2).

### Optimal modified STarT Back Tool score cut-offs in both data sets

Table 1 reports sensitivity, specificity and the Youden's J statistic for each possible modified STarT Back Tool score cut-point at baseline for each pain region. The results demonstrate that the optimal STarT Back Tool baseline score cut-point for discriminating ‘poor outcome’ at 6-month follow-up was not consistent across pain regions. For example, among (PhysioDirect) patients with neck, back and multisite pain, the optimal STarT Back Tool cut-point for discriminating ‘poor outcome’ was 5, whereas this was 4 for those with upper limb and lower limb as their primary pain site.

**Table 1:** Identifying optimal modified STarT Back Tool cut-points for each pain region using (1) Youden's J statistic and (2) a clinically defined maximum specificity of 0.7

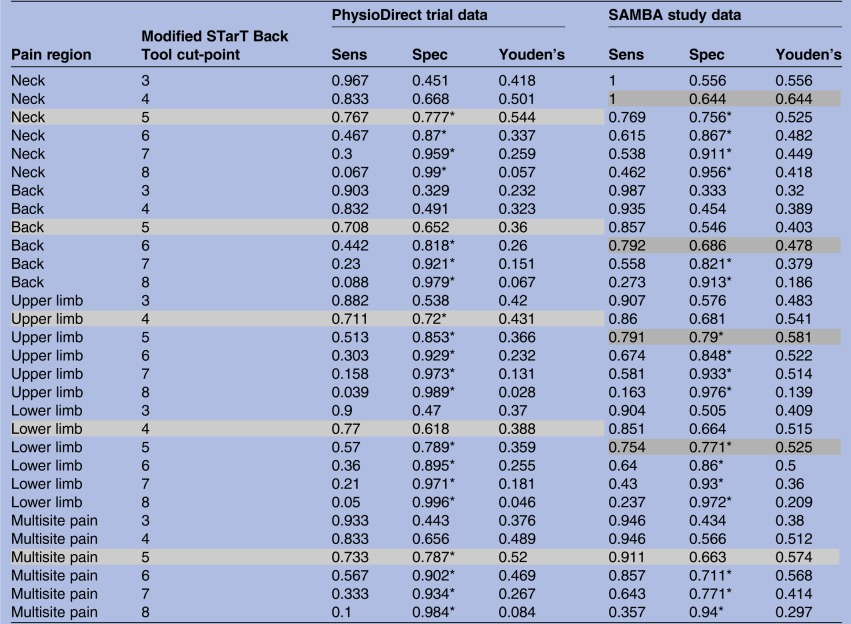

## Discussion

This is the first study to demonstrate that a modified STarT Back Tool is similarly predictive of 6-month physical health (defined by worst tertile of the SF-36) across different musculoskeletal pain regions. Predictive performance determined by AUCs for the 8-item modified STarT Back Tool total score was in fact slightly higher for neck, upper limb, lower limb and multisite pain than for back pain, although differences were not statistically significant. The results therefore demonstrate that the prognostic factors included within the STarT Back Tool are predictive of 6-month physical health across a range of musculoskeletal pain regions, not just back pain. However, the results demonstrated that the optimal baseline STarT Back Tool score cut-point for identifying individuals with poor physical health outcome was neither consistent across different pain regions nor across clinical services (community physiotherapy services (PhysioDirect trial)) and primary–secondary care interface services (SAMBA study). This finding was consistent regardless of method used to determine the optimal modified STarT Back Tool score cut-point (Youden's J statistic or an a priori defined maximum false-positive rate of 30%). This implies that the existing original STarT Back Tool score cut-point (4 or more out of 9) used to allocate patients with low back pain to the medium-risk/high-risk subgroups cannot simply be applied to patients with other musculoskeletal pain presentations or in different clinical services. This is likely to be due to differences in patient characteristics across services such as episode duration, which is known to influence the performance of the original STarT Back Tool.^33^ It is also likely that individual modified STarT Back Tool items are not equally applicable to patients with pain in the five regions.^34^ For example, the item about walking difficulties is likely to be less relevant and therefore less predictive of physical health outcome for patients with upper limb pain than for those with lower limb or spinal pain. A key message from this study is the value and importance of testing the capabilities of the STarT Back Tool in different settings and patient populations and not presuming that existing primary care subgroup cut-points will be the same in other groups. If wider validity is demonstrated, this will help strengthen the case for the general applicability of the tool.

The findings of this study concur with previous evidence suggesting that the same set of prognostic variables can be used to estimate prognosis of patients with different musculoskeletal pain presentations.^7^ ^15^ ^17^ The STarT Back Tool uses biopsychosocial constructs known to predict persistent disability among patients with low back pain, such as: difficulty with walking and dressing, pain elsewhere, fear avoidance, pain catastrophising, anxiety and low mood.^1^ However, the STarT Back Tool is not just a prognostic index, but is used to stratify patients for different matched treatments. An important issue highlighted by this analysis is that if clinicians simply modify the STarT Back Tool for use with other musculoskeletal pain patients, they are at risk of matching patients to inappropriate treatments. It is also apparent that future translation and validation studies of the STarT Back Tool need to carefully consider adopting the same STarT Back Tool score cut-points as used in the original UK STarT Back Tool study^1^ without first testing if these cut-points are appropriate for their own clinical populations. Based on these findings, our team has begun to further refine and validate an improved stratification tool—the Keele STarT MSK Tool—which will be specifically designed for use with primary care patients consulting with the five most common musculoskeletal pain presentations in a new programme of research. While our study was not able to examine optimal high-risk subgroup cut-offs for ‘distressed’ patients, a previous cross-sectional study^34^ in a US physical therapy population has compared the relationships between a modified STarT Back Tool and psychological measures in people with different pain regions. It is found that regardless of body region of pain, higher modified STarT Back Tool scores were associated with higher levels of kinesiophobia, catastrophising, fear avoidance, anxiety and depressive symptoms. The strengths of our analyses reported here include the large sample sizes of the PhysioDirect and SAMBA studies and the opportunity to examine optimal cut-points in patients with different pain sites and in different NHS musculoskeletal services. An additional strength was that both studies used the same measure of physical health (SF-36), had the same 6-month follow-up time-point and included patients whose pain could be classified into the same musculoskeletal pain regions. Given the potential weakness of using the Youden's J Statistic to define optimal cut-points for discriminating between low and medium/high risk, we also used a clinically determined guide (maximum false-positive rate), which showed similar inconsistencies in optimal cut-off between regional pain site and clinical setting. One weakness is that the original STarT Back Tool was not included in these two data sets, which meant a direct comparison between the performance of the original and modified versions for patients with low back pain was not possible. The choice of poor physical health outcome at 6 months using the lowest tertile on the SF-12 was also relatively arbitrary, but served the purpose of this analysis to compare outcome between different regional pain sites, making the exact definition of poor outcome less critical to the study aims. It should be noted that the different levels of poor clinical outcome between the PhysioDirect (18.5%) and Samba (28.2%) studies could be due to the different settings and design of these two studies and it is possible this may have influenced the findings.

The implications from this analysis are that, despite good predictive performance of the modified STarT Back Tool in patients with pain in different regions of the body, clinicians need to cautiously consider the choice of cut-points when using a modified STarT Back Tool for musculoskeletal pain regions other than low back pain. The results suggest that existing cut-points may lead to an inefficiency in healthcare resource use, with too many patients with a likely long-term disability being missed, or too many patients with good physical health outcome being inaccurately classified as ‘at risk’, which may result in over treatment of low-risk groups.

## Conclusions

A modified version of the STarT Back Tool has similar predictive performance when used for patients with musculoskeletal pain in different body regions. However, the cut-points used to identify patients with a poor physical health outcome at 6-month follow-up are not consistent across pain regions or clinical services. Further research is underway to refine and validate a new Keele STarT MSK Tool which will form part of a new stratified care approach to be tested in a randomised controlled trial.
